# The Association of Mid-Regional Pro-Adrenomedullin and Mid-Regional Pro-Atrial Natriuretic Peptide with Mortality in an Incident Dialysis Cohort

**DOI:** 10.1371/journal.pone.0017803

**Published:** 2011-03-07

**Authors:** Ghazaleh Gouya, Gisela Sturm, Claudia Lamina, Emanuel Zitt, Otto Freistätter, Joachim Struck, Michael Wolzt, Florian Knoll, Friederike Lins, Karl Lhotta, Ulrich Neyer, Florian Kronenberg

**Affiliations:** 1 Department of Clinical Pharmacology, Medical University Vienna, Vienna, Austria; 2 Division of Genetic Epidemiology, Department of Medical Genetics, Molecular and Clinical Pharmacology, Innsbruck Medical University, Innsbruck, Austria; 3 Department of Nephrology and Dialysis, Academic Teaching Hospital Feldkirch, Feldkirch, Austria; 4 Vorarlberg Institute for Vascular Investigation and Treatment (VIVIT), Feldkirch, Austria; 5 Research Department, B.R.A.H.M.S GmbH (Part of ThermoFisher Scientific), Hennigsdorf/Berlin, Germany; Innsbruck Medical University, Austria

## Abstract

High levels of the plasma peptides mid-regional pro-adrenomedullin (MR-proADM) and mid-regional pro-atrial natriuretic peptide (MR-proANP) are associated with clinical outcomes in the general population. Data in patients with chronic kidney disease are sparse. We therefore investigated the association of MR-proANP and MR-proADM levels with all-cause and cardiovascular (CV) mortality, CV events and peripheral arterial disease in 201 incident dialysis patients of the INVOR-Study prospectively followed for a period of up to more than 7 years. The overall mortality rate was 43%, thereof 43% due to CV events. Both baseline MR-proANP and MR-proADM were associated with higher risk of all-cause (HR = 1.44, p = 0.001 and HR = 1.32, p = 0.002, respectively) and CV mortality (HR = 1.75, p<0.001 and HR = 1.41, p = 0.007, respectively) after adjustment for age, sex, previous CV events, diabetes mellitus and time-dependent type of renal replacement therapy. We then stratified patients in high risk (both peptides in the upper tertile), intermediate risk (only one of the two peptides in the upper tertile) and low risk (none in the upper tertile). Although demographic, clinical and laboratory variables were similar among the intermediate and high risk group, to be with both parameters in the upper tertile was associated with a 3-fold higher risk for all-cause (HR = 2.87, p<0.001) and CV mortality (HR = 3.58, p = 0.001). In summary, among incident dialysis patients MR-proANP and MR-proADM were shown to be associated with all-cause and CV mortality, with the highest risk when both parameters were in the upper tertiles.

## Introduction

Patients with chronic kidney disease (CKD) are at increased risk for death. Cardiovascular and non-cardiovascular mortality are 8 to 9 times higher in incident dialysis patients when compared to the general population [1]. Biomarkers which predict these fatal outcomes and provide insight into the pathogenesis are poorly established but highly required for an early risk stratification of this high risk cohort of patients [2], [3].

There is evidence that peptides involved in maintaining the cardiovascular and renal homeostasis such as atrial natriuretic peptide (ANP) and adrenomedullin (ADM) may play a key role in the compensatory mechanisms of CKD. Both hormones are elevated in the early stages of CKD [4], [5] and were shown to be highly predictive for progression of CKD in nondiabetic patients [6], [7]. Elevated levels of ANP [8]–[12] and ADM [13] have been reported to be associated with cardiac events, overall and cardiovascular mortality in patients already on dialysis. Increased levels of ANP and ADM have been associated with higher mortality rates in cohorts of cardiac patients as well as in patients with type 2 diabetes mellitus [14]–[17]. The two peptides might provide additional help to estimate volume status and are probably also an early alarm signal of deteriorating hemodynamic changes in CKD patients. Since both peptides increase with deteriorating kidney function [6], it is unclear whether recently established thresholds [18] can be applied for prediction of all-cause and cardiovascular mortality in patients with renal disease.

The aim of this study was therefore to investigate whether MR-proANP and MR-proADM plasma concentrations at the start of dialysis treatment are associated with risk of cardiovascular or all-cause mortality in a prospective cohort study of incident dialysis patients.

## Methods

### INVOR-Study

The INVOR-Study [19] (Study of Incident Dialysis Patients in Vorarlberg) is a single-center, prospective, observational cohort study of incident Caucasian hemodialysis and peritoneal dialysis patients in Vorarlberg, the westernmost province of Austria counting approximately 400,000 inhabitants.

Ethic statement: The study was approved by the ethics committee of the Innsbruck Medical University and all patients enrolled in the study provided written informed consent.

All incident dialysis patients from this province starting chronic dialysis treatment between May 1^st^, 2000 and April 30^th^, 2006 were consecutively enrolled with the advantage that all patients of this region are treated by the same care provider. During this period of 6 years a total number of 235 incident dialysis patients were included and followed until the study endpoint was reached or follow-up was censored at December 31^st^, 2009. Ten patients having a malignant tumor at initiation of dialysis were not recruited defined by the exclusion criteria. Due to inappropriate or missing blood samples MR-proANP and MR-proADM was measured in 201 out of 235 patients. All data and analyses described in this manuscript are based on these 201 patients.

### Data description

Clinical, laboratory and medication data were collected prospectively starting at the time of initiation of dialysis. These data included age, sex, height, weight, body mass index, diabetes status and current smoking status. Type of and change in renal replacement therapy (hemodialysis, peritoneal dialysis and kidney transplantation) were recorded and considered as time-dependent treatment status for data analysis. Vascular access procedures and the type of vascular access (native fistula, graft or central venous catheter) for hemodialysis were also evaluated.

Information on the following clinical events were collected before initiation of dialysis and during the entire observation period thereafter: coronary artery disease (including myocardial infarction, percutaneous transluminal coronary angioplasty, aortocoronary bypass), cardiovascular disease (including myocardial infarction, percutaneous transluminal coronary angioplasty, aortocoronary bypass, angiographically-proven coronary stenosis ≥50%, sudden cardiac death, ischemic or hemorrhagic cerebral infarction, transient ischemic attack, carotid stenosis and carotid endarterectomy), peripheral arterial disease (significant ultrasound- or angiographically-proven vascular stenosis, percutaneous transluminal angioplasty, peripheral bypass, amputation).

### Biochemical analysis

We used two novel commercially available fully automated sandwich immunoassays for the measurement of MR-proANP (B.R.A.H.M.S MR-proANP KRYPTOR) and MR-proADM (B.R.A.H.M.S MR-proADM KRYPTOR) according to the manufacturer's instruction manuals (B.R.A.H.M.S GmbH, Hennigsdorf Germany). The design of these assays is based on immunoluminometric assays described previously [20], [21]. Both parameters were measured in plasma collected at the time immediately before the initiation of the first dialysis therapy and kept frozen at −80°C until measurement in a single batch. Other laboratory parameters reported here were measured from the same blood collection immediately before the first dialysis therapy.

As described earlier, the limit of quantitation was 4.5 pmol/L for the MR-proANP assay with a within-run imprecision coefficient of variation (CV) of <4.5% between 10 and 20 pmol/L and <2.5% between 20 and 1000 pmol/L, and between-run imprecision CV of <6.5% between 10 and 1000 pmol/L [21]. The limit of quantification was 0.23 nmol/L for the MR-proADM assay with a within-run imprecision CV of <4% between 0.5 and 2 nmol/L and <2% between 2 and 6 nmol/L, and a between-run imprecision CV of <11% between 0.5 and 2 nmol/L and <10% between 2 and 6 nmol/L [20]. All blood samples were processed by personnel blinded from any patient data.

### Study Outcomes

The first outcome of interest was all-cause mortality and cardiovascular mortality. We also investigated other endpoints such as cardiovascular disease and peripheral arterial disease. Cardiovascular mortality was defined as death of myocardial infarction, heart failure, sudden death, ischemic or hemorrhagic stroke. Cardiovascular disease (CVD) events were defined as fatal and non-fatal myocardial infarction, percutaneous transluminal coronary angioplasty (PTCA), aortocoronary bypass (ACBP), angiographically-proven coronary stenosis ≥50%, ischemic cerebral infarction or transient ischemic attack. For peripheral arterial disease (PAD) at least one of the following events was existent: significant ultrasound- or angiographically-proven vascular stenosis, percutaneous transluminal angioplasty (PTA), peripheral bypass or amputation. An incident PAD event was only considered as a first time manifestation or a deterioration of PAD in terms of e.g. a change in PAD stage according to Fontaine. Two patients were lost to follow-up, one regained renal function and the other one moved away.

### Statistical Methods

At baseline, categorical data were compared using χ^2^-test and continuous variables were analyzed using an unpaired t-test or the non-parametric Wilcoxon rank-sum test. Data are presented as mean±SD and as median and 25^th^ and 75^th^ percentiles for skewed variables where appropriate.

To investigate the influence of MR-proANP and MR-proADM on all-cause mortality, cardiovascular mortality, cardiovascular disease and peripheral arterial disease, multivariable adjusted Cox-proportional hazards regression models were performed. In all analyses a p-value of 0.05 was considered significant. Variables were chosen for the multiple Cox regression analysis since they were either well-known risk factors for all-cause or CV mortality or they showed correlations with MR-proADM or MR-proANP. The first model was adjusted for age, sex, previous cardiovascular events, diabetes mellitus and type of renal replacement therapy, which was modeled time-dependently (Model 1). The second model (Model 2) was additionally adjusted for albumin, C-reactive protein, current smoking status, native fistula and echocardiographic data (ejection fraction ≤60% versus >60%). Due to sparseness of the data and risk of overfitting, this fully-adjusted model should only be considered as a sensitivity analysis to Model 1, which is taken as the main model in this investigation. Linear relationship assumption of MR-proANP and MR-proADM in the Cox models was tested, as well as the proportional hazards assumption. One standard deviation (SD) was taken as the unit of measure for each of the continuous outcome variables to ensure comparability of Hazard Ratios. For ease of interpretation, additional fully adjusted Cox-proportional hazards regression models were calculated by dividing MR-proANP and MR-proADM into tertiles and also by stratifying patients in groups of high risk (both MR-proANP and MR-proADM in the upper tertile), intermediate risk (only one of the two parameters in the upper tertile) and low risk (none of the two parameters in the upper tertile). Adjusted survival curves are given for each of these risk groups and for each of the tertiles, holding all covariates fixed at their mean level. We also performed a sensitivity analysis with censoring at the time of transplantation. All analyses were conducted in SPSS version 16.0 software (SPSS Inc., Chicago, IL, USA) and R using the package “survival”.

## Results

**Figure 1:**
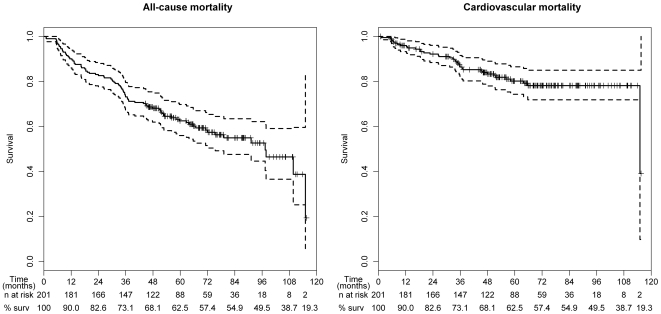
Kaplan-Meier survival curves with 95% confidence bands for all-cause and cardiovascular mortality. “% surv” stands for the percentage of survivors at each 12-month interval.

Table 1 provides an overview on the demographic and laboratory parameters of our study cohort including MR-proANP, and MR-proADM plasma concentrations. Causes of CKD were diabetic nephropathy (32%), vascular nephropathy (25%), glomerulonephritis (15%), interstitial and reflux nephropathy (9%), polycystic kidney disease (9%) and other causes (10%). During the entire observation period of up to more than 7 years 86 of the 201 patients (43%) passed away, 43% of them due to cardiovascular events (Figure 1). Patients who did not survive were older and had more severe illness compared to their event-free counterparts as indicated by lower serum albumin levels and impaired left ventricular systolic function, a higher frequency of diabetes as well as higher prevalence of comorbidities such as coronary and peripheral artery disease at baseline when compared to survivors. Almost all patients (99%) had MR-proANP levels ≥120 pmol/L, a recently investigated cut point for diagnosis of acute heart failure in patients with acute dyspnea [18]. 82% of all patients had at baseline MR-proADM levels above 1.985 nmol/L which was recently shown as optimal cut point to predict all-cause mortality in patients with acute dyspnea [18]. MR-proANP and MR-proADM levels were significantly higher among the non-survivors compared to the survivors (981±573 pmol/L vs. 662±439 pmol/L, p<0.001 and 3.29±1.17 nmol/L vs. 2.72±1.33 nmol/L, p<0.001, respectively). Table 2 lists the correlations between MR-proANP, MR-proADM and relevant parameters. Both parameters were moderately correlated with each other (r^2^ = 0.62).

**Table 1:** Clinical characteristics of patients.

	All patients(n = 201)	Survivors(n = 115)	Non-Survivors(n = 86)
Sex (male/female), n (%)	124/77 (62/38%)	69/46 (60/40%)	55/31 (64/36%)
Age (years)	61±14	56±15	69±10^d^
Diabetes mellitus, n (%)	75 (37%)	29 (25%)	46 (54%)^d^
Current smokers, n (%)	44 (22%)	29 (25%)	15 (17%)
Body Mass Index (kg/m^2^)	26.1±4.4	25.9±4.4	26.2±4.5
Start of dialysis with			
Hemodialysis, n (%)	169 (84%)	93 (81%)	76 (88%)
Central venous catheter, n (%)	25 (15%)	8 (9%)	17 (22%)^a^
Native fistula, n (%)	114 (67%)	73 (79%)	41 (54%)^c^
Graft, n (%)	30 (18%)	12 (13%)	18 (24%)
Peritoneal dialysis, n (%)	32 (16%)	22 (19%)	10 (12%)
Year of start of dialysis			
2000–2003, n (%)	98 (49%)	51 (44%)	47 (55%)
2004–2006, n (%)	103 (51%)	64 (56%)	39 (45%)
Echocardiography			
Missing, n (%)	15 (7%)	5 (4%)	10 (12%)
Ejection fraction ≤60%, n (%)	86 (43%)	42 (37%)	44 (51%)^b^
Ejection fraction >60%, n (%)	100 (50%)	68 (59%)	32 (37%)^b^
Systolic blood pressure (mmHg)	153±23	154±22	153±24
Diastolic blood pressure (mmHg)	83±12	86±11	79±13^d^
***Laboratory parameters***			
MR-proANP (pmol/L)	798±524[461; 669; 946]	662±439[326; 571; 787]	981±573^d^[544; 786; 1405]
MR-proADM (nmol/L)	2.97±1.29[2.20; 2.69; 3.52]	2.72±1.33[2.05; 2.49; 3.13]	3.29±1.17^d^[2.49; 3.02; 3.88]
Albumin (g/dL)	3.7±0.8	3.9±0.8	3.5±0.6^d^
C-reactive protein (mg/dL)	3.0±5.4[0.3; 0.8; 2.5]	2.6±4.3[0.3; 0.7; 2.7]	3.8±6.5^a^[0.5; 1.0; 2.5]
Calcium (mmol/L)	2.12±0.28	2.16±0.27	2.08±0.28^a^
Phosphorus (mmol/L)	2.00±0.61[1.57; 1.94; 2.33]	1.96±0.61[1.51; 1.90; 2.25]	2.04±0.61[1.60; 2.00; 2.39]
Hemoglobin (g/dL)	11.2±1.7	11.5±1.7	10.8±1.6^b^
Creatinine (mg/dL)	7.3±2.7[5.5; 6.8; 8.7]	7.3±2.5[5.5; 6.8; 8.7]	7.3±2.9[5.3; 6.8; 8.4]
HbA1c (%)	6.43±1.55	6.15±1.32	6.70±1.71
Total cholesterol (mg/dL)	189±52	190±49	187±55
LDL cholesterol (mg/dL)	118±44	121±44	114±42
HDL cholesterol (mg/dL)	46.4±13.4	47.8±13.4	44.5±13.1
Triglycerides (mg/dL)	166±102[106; 139; 192]	164±86[106; 139; 193]	169±120[105; 137; 189]
***Comorbidities before dialysis***			
CAD*, n (%)	36 (17.9%)	13 (11.3%)	23 (26.7%)^b^
CVD**, n (%)	61 (30.3%)	23 (20.0%)	38 (44.2%)^d^
PAD***, n (%)	35 (17.4%)	8 (7.0%)	27 (31.4%)^d^
***Follow-up***			
Follow-up time (months)‡	55.7±28.7	71.3±19.2	34.8±26.0^d^
Transplantation, n (%)	59 (29.4%)	53 (46.1%)	6 (7.0%)^d^

Mean±SD [25., 50. und 75. percentile in case of non-normal distribution] or number (%).

a p<0.05;

b p<0.01;

c p<0.005;

d p<0.001 – comparison between survivors and non-survivors.

*Coronary artery disease (CAD): myocardial infarction, percutaneous transluminal coronary angioplasty, aortocoronary bypass.

**Cardiovascular disease (CVD): myocardial infarction, percutaneous transluminal coronary angioplasty, aortocoronary bypass, angiographically-proven coronary stenosis ≥50%, ischemic cerebral infarction, transient ischemic attack.

***Peripheral arterial disease (PAD): significant ultrasound- or angiographically-proven vascular stenosis, percutaneous transluminal angioplasty, peripheral bypass, amputation.

‡ Follow-up time was calculated as the time from the start of dialysis until the patient died or the end of the observation period was reached.

**Table 2:** Correlations between mid-regional pro-atrial natriuretic peptide (MR-proANP) and mid-regional pro-adrenomedullin (MR-proADM) and different parameters.

	Correlation coefficient (r)	
	MR-proANP	MR-proADM
Sex (male/female)	−0.032	−0.042
Age (years)	0.368^c^	0.298^c^
Diabetes mellitus (no/yes)	0.227^b^	0.091
Current smokers (no/yes)	−0.084	0.016
Body Mass Index (kg/m^2^)	−0.090	0.157^a^
Left ventricular ejection fraction (≤60%/>60%)	−0.262^c^	−0.257^c^
Systolic blood pressure (mmHg)	0.133	0.056
Diastolic blood pressure (mmHg)	−0.078	−0.082
***Laboratory parameters***		
Albumin (g/dL)	−0.198^b^	−0.223^b^
C-reactive protein (mg/dL)	0.003	0.084
Hemoglobin (g/dL)	−0.164^a^	−0.223^b^
Creatinine (mg/dL)	−0.070	0.032
***Comorbidities before dialysis***		
CAD*	0.168^a^	0.082
CVD**	0.150^a^	0.075
PAD***	0.168^a^	0.101

For footnotes see Table 1.

a p<0.05;

b p<0.01;

c p<0.001.

### Cox-proportional hazards models for continuous variables of MR-proANP and MR-proADM

Table 3 presents the results from Cox-proportional hazards models for MR-proANP and MR-proADM, and all-cause and cardiovascular mortality as well as CVD and PAD events. Hazards ratios are adjusted for age, sex, previous CVD events, diabetes mellitus and time-dependent type of renal replacement therapy (Model 1) and an extended adjustment additionally for albumin, CRP, current smoking, native fistula and ejection fraction (Model 2 as sensitivity analysis). On a continuous scale, both MR-proANP and MR-proADM were significantly associated with all-cause and with cardiovascular mortality but not with the entire group of fatal and non-fatal cardiovascular disease events. When PAD was used as outcome variable only plasma MR-proANP but not MR-proADM levels showed a significant association. These results were only marginally influenced if further adjusted for albumin, CRP, current smoking, native fistula and ejection fraction determined by echocardiography (Model 2).

**Table 3:** The association of MR-proANP and MR-proADM as well as MR-proANP tertiles and MR-proADM tertiles and furthermore for patients with high risk (both MR-proANP and MR-proADM in the highest tertile) with different endpoints using multiple Cox-proportional hazards models.

	All-cause mortality			Cardiovascular mortality*			Cardiovascular disease**			Peripheral arterial disease***		
	(n events = 86)			(n events = 37)			(n events = 85)			(n events = 54)		
	HR	95%CI	p-value	HR	95%CI	p-value	HR	95%CI	p-value	HR	95%CI	p-value
**MR-proANP (per 1 SD increase)** a												
Model 1	1.44	(1.17, 1.78)	0.001	1.75	(1.28, 2.39)	<0.001	1.15	(0.92, 1.45)	0.221	1.34	(1.02, 1.77)	0.037
Model 2	1.32	(1.04, 1.68)	0.021	1.73	(1.23, 2.44)	0.002	1.06	(0.83, 1.36)	0.642	1.35	(0.99, 1.84)	0.058
**MR-proADM (per 1 SD increase)** a												
Model 1	1.32	(1.11, 1.58)	0.002	1.41	(1.10, 1.82)	0.007	1.17	(0.98, 1.39)	0.092	1.08	(0.83, 1.41)	0.556
Model 2	1.23	(1.00, 1.50)	0.051	1.43	(1.07, 1.91)	0.015	1.11	(0.90, 1.36)	0.331	1.08	(0.80, 1.46)	0.603
**MR-proANP (tertiles)** b												
≤522 pmol/L	1			1			1			1		
523–794 pmol/L	1.07	(0.55, 2.05)	0.847	1.55	(0.50, 4.77)	0.446	0.97	(0.53, 1.78)	0.931	0.90	(0.40, 2.02)	0.802
≥795 pmol/L	1.76	(0.93, 3.33)	0.082	2.96	(0.99, 8.89)	0.053	1.07	(0.57, 1.98)	0.839	1.10	(0.49, 2.46)	0.815
**MR-proADM (tertiles)** b												
≤2.40 nmol/L	1			1			1			1		
2.41–3.10 nmol/L	1.04	(0.56, 1.92)	0.909	1.09	(0.41, 2.90)	0.862	1.03	(0.59, 1.80)	0.910	1.16	(0.59, 2.27)	0.674
≥3.11 nmol/L	2.39	(1.33, 4.28)	0.003	3.16	(1.27, 7.83)	0.013	1.53	(0.88, 2.67)	0.130	1.40	(0.68, 2.85)	0.360
**MR-proANP- MR-proADM- Score** b												
Low & intermediate risk	1			1			1			1		
High risk	2.87	(1.77, 4.65)	<0.001	3.58	(1.73, 7.43)	0.001	1.45	(0.87, 2.44)	0.156	1.59	(0.85, 2.97)	0.147

MR-proANP, mid-regional pro-atrial natriuretic peptide; MR-proADM, mid-regional pro-adrenomedullin.

**Model 1**: adjusted for age, sex, previous CVD**, diabetes mellitus, time-dependent type of renal replacement therapy.

**Model 2**: adjusted as in model 1 and additionally for albumin, CRP, current smoking, native fistula, echocardiography (ejection fraction ≤60% and >60%).

a For MR-proANP and MR-proADM 1 standard deviation (SD) increment was 524 pmol/L and 1.29 nmol/L, respectively. One SD was taken as the unit of increment for each of the continuous outcome variables to ensure comparability of Hazard Ratios.

b Adjusted for age, sex, previous CVD**, diabetes mellitus, time-dependent type of renal replacement therapy.

*CV mortality: myocardial infarction, heart failure, sudden cardiac death, ischemic stroke, hemorrhagic stroke.

**CVD: myocardial infarction, percutaneous transluminal coronary angioplasty, aortocoronary bypass, angiographically-proven coronary stenosis ≥50%, ischemic or hemorrhagic cerebral infarction, transient ischemic attack, carotid stenosis and carotid endarterectomy.

***PAD: significant ultrasound- or angiographically-proven vascular stenosis, percutaneous transluminal angioplasty, peripheral bypass, amputation.

### Cox-proportional hazards models for categorical analysis of MR-proANP and MR-proADM

**Figure 2:**
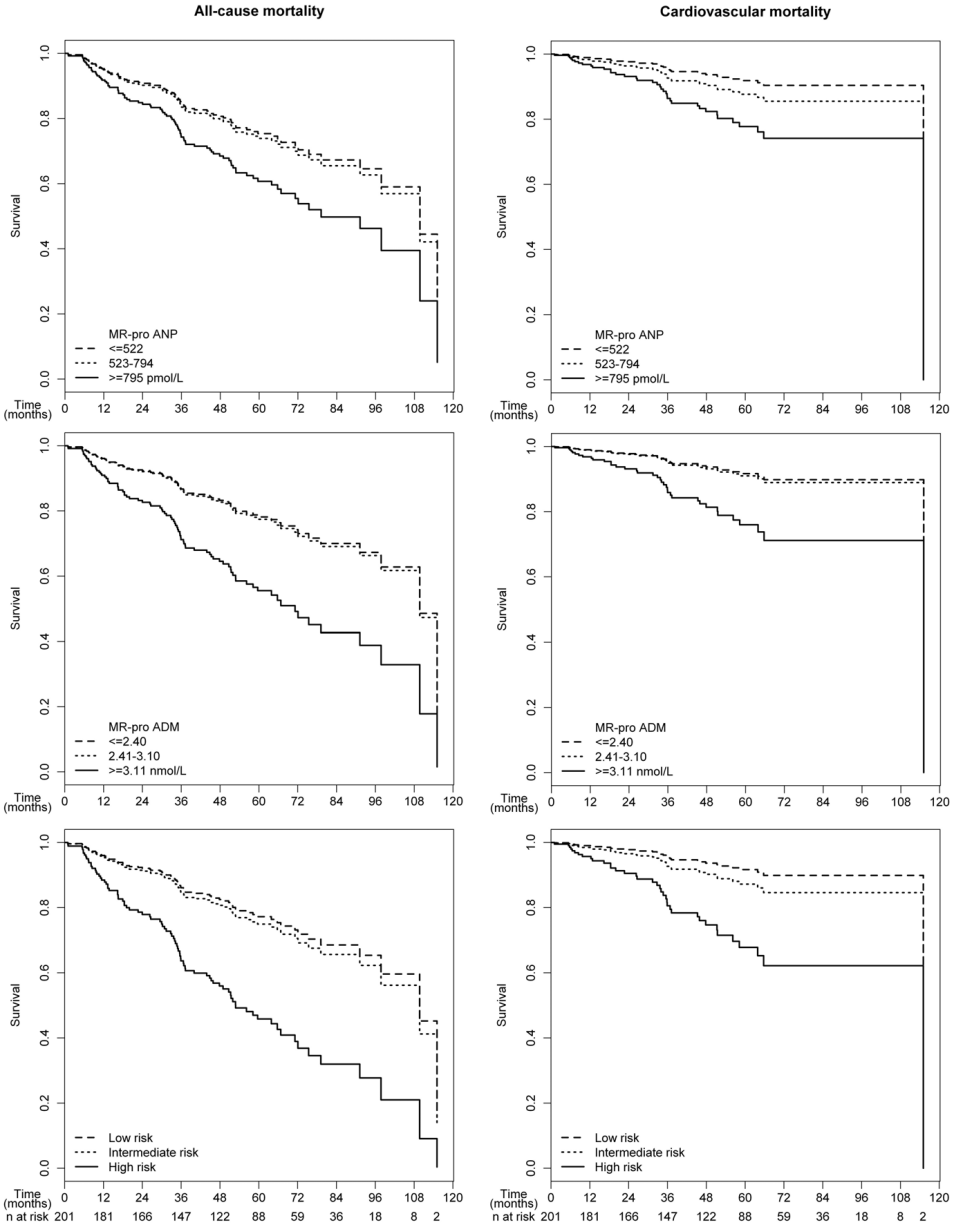
Survival curves for all-cause and cardiovascular mortality adjusted for age, sex, previous cardiovascular events, diabetes mellitus and time-dependent type of renal replacement therapy for mid-regional pro-atrial natriuretic peptide (MR-proANP) tertiles (top), mid-regional pro-adrenomedullin (MR-proADM) tertiles (middle) and patients stratified for high risk (both MR-proANP and MR-proADM in the upper tertile), intermediate risk (only one of the two parameters in the upper tertile) and low risk (none of the two parameters in the upper tertile) (bottom). Tertiles for MR-proANP were ≤522, 523–794, and ≥795 pmol/L, respectively. Tertiles for MR-proADM were ≤2.40, 2.41–3.10, and ≥3.11 nmol/L, respectively.

When we analyzed the data according to tertiles of MR-proANP and MR-proADM (Table 3), a non-significant trend for the upper tertile of MR-proANP for all-cause (HR = 1.76, p = 0.082) and cardiovascular mortality (HR = 2.96, p = 0.053) was observed. However, patients in the upper tertile of MR-proADM had a significantly increased risk for all-cause (HR = 2.39, p = 0.003) as well as cardiovascular mortality (HR = 3.16, p = 0.013). Survival curves for all-cause and cardiovascular mortality demonstrated that the first and second tertile of MR-proANP and MR-proADM were similar and only the third tertile discriminated between survivors and non-survivors (Figure 2).

This increased risk was clearly identified using a stratification based on the tertiles of the two peptides by combining the highest tertiles of MR-proANP (≥794 pmol/L) and MR-proADM (≥3.11 nmol/L). Table 4 provides the demographic and clinical characteristics of patients stratified in high risk (both MR-proANP and MR-proADM in the upper tertile), intermediate risk (only one of the two parameters in the upper tertile) and low risk (none of the two parameters in the upper tertile). The increase in risk with respect to all-cause mortality was almost identical when we compared the high risk group versus intermediate risk group (HR = 2.70, p = 0.001) and the high risk group versus low risk group (HR = 3.02, p<0.001). The risk was only increased when a patient had both parameters in the upper tertile, although demographic, clinical and laboratory variables were similar among the intermediate and high risk group. The high risk group was associated with an about 3-fold higher risk for all-cause mortality (HR = 2.87, p<0.001) and cardiovascular mortality (HR = 3.58, p = 0.001) when compared to the remaining patients (Table 3 and Figure 2).

**Table 4:** Clinical characteristics of patients stratified in high risk (both MR-proANP and MR-proADM in the upper tertile), intermediate risk (only one of the two parameters in the upper tertile) and low risk (none of the two parameters in the upper tertile).

	Low risk(n = 107)	Intermediate risk(n = 51)	High risk(n = 43)
Sex (male/female), n (%)	69/38 (64/36%)	32/19 (63/37%)	23/20 (53/47%)
Age (years)	57±15	67±11	66±11
Diabetes mellitus, n (%)	34 (32%)	22 (43%)	19 (44%)
Current smokers, n (%)	26 (24%)	9 (18%)	9 (21%)
Body Mass Index (kg/m^2^)	26.0±4.1	26.6±4.4	25.5±5.4
Start of dialysis with			
Hemodialysis, n (%)	81 (76%)	49 (96%)	39 (91%)
Central venous catheter, n (%)	9 (11%)	9 (18%)	7 (18%)
Native fistula, n (%)	58 (72%)	32 (65%)	24 (62%)
Graft, n (%)	14 (17%)	8 (16%)	8 (21%)
Peritoneal dialysis, n (%)	26 (24%)	2 (4%)	4 (9%)
Year of start of dialysis			
2000–2003, n (%)	55 (51%)	26 (51%)	17 (40%)
2004–2006, n (%)	52 (49%)	25 (49%)	26 (60%)
Echocardiography			
Missing, n (%)	5 (5%)	4 (8%)	6 (14%)
Ejection fraction ≤60%, n (%)	36 (34%)	27 (53%)	23 (53%)
Ejection fraction >60%, n (%)	66 (62%)	20 (39%)	14 (33%)
Systolic blood pressure (mmHg)	151±22	154±21	157±25
Diastolic blood pressure (mmHg)	84±12	81±11	82±15
***Laboratory parameters***			
MR-proANP (pmol/L)	488±188[322; 512; 625]	905±521[546; 788; 1055]	1443±455[975; 1422; 1811]
MR-proADM (nmol/L)	2.19±0.55[1.84; 2.31; 2.57]	3.28±0.78[2.71; 3.15; 3.69]	4.51±1.54[3.46; 4.15; 4.93]
Albumin (g/dL)	3.8±0.6	3.7±0.6	3.5±0.7
C-reactive protein (mg/dL)	2.6±4.5[0.3; 0.8; 2.1]	4.3±7.4[0.3; 0.9; 4.4]	2.9±4.3[0.4; 1.6; 3.1]
Calcium (mmol/L)	2.18±0.28	2.08±0.28	2.05±0.24
Phosphorus (mmol/L)	2.02±0.62[1.59; 1.95; 2.40]	1.90±0.54[1.54; 1.90; 2.14]	2.04±0.66[1.57; 1.97; 2.39]
Hemoglobin (g/dL)	11.5±1.7	11.1±1.6	10.5±1.8
Creatinine (mg/dL)	7.3±2.7[5.5; 6.8; 8.7]	7.4±2.5[5.8; 7.1; 8.1]	7.2±2.9[5.3; 6.4; 8.5]
HbA1c (% Hb)	6.51±1.44	6.57±2.02	6.16±1.21
Total cholesterol (mg/dL)	192±54	192±52	173±44
LDL cholesterol (mg/dL)	122±46	120±43	107±38
HDL cholesterol (mg/dL)	46.2±14.2	44.8±13.0	49.2±11.1
Triglycerides (mg/dL)	170±90[111; 143; 212]	178±136[109; 139; 194]	142±76[100; 121; 161]
***Comorbidities before dialysis***			
CAD*, n (%)	15 (14%)	12 (23.5%)	9 (20.9%)
CVD**, n (%)	28 (26%)	19 (37.3%)	14 (32.6%)
PAD***, n (%)	18 (17%)	5 (9.8%)	12 (27.9%)
***Follow-up***			
Follow-up time (months)**‡**	63.2±26.6	56.3±28.5	36.1±25.3
All-cause mortality, n (%)	32 (30%)	26 (51%)	28 (65%)
Transplantation, n (%)	44 (41%)	8 (15.7%)	7 (16.3%)

For footnotes see Table 1.

### Sensitivity analysis

It might introduce some considerable bias to the data analysis when a patient is selected for transplantation and follow-up time is censored at the time of transplantation. For this reason the above main analysis was performed by a time-dependent modeling of the renal replacement therapy status as discussed earlier [22], [23]. To exclude that this procedure has influenced our main findings, we performed a sensitivity analysis with classical censoring at the time of transplantation which did, however, not reveal any substantial differences in HRs compared to the primary analysis (see Table S1). Due to sparseness of the data and risk of overfitting we also calculated a simple model only adjusting for age and sex that did not show major differences in estimates compared to Model 1. A further sensitivity analysis was calculated where Model 1 was additionally adjusted for hemoglobin. No substantial differences in estimates could be observed.

## Discussion

The study at hand investigated the two peptides MR-proANP and MR-proADM in a prospective long-term cohort study of incident dialysis patients. We observed that increased concentrations of both peptides are associated with an increased risk of all-cause as well as cardiovascular mortality and this risk was about threefold elevated when both parameters were in the upper tertile of the entire patient group. This elevation of both parameters discriminated especially patients with intermediate and high risk which were otherwise similar in terms of clinical and laboratory parameters. Therefore, the incremental benefit to identify high risk patients is achieved by the combined evaluation of these two biomarkers. If a patient had an isolated increase in one of the two parameters, the risk was similar compared to patients which did show an increase at all (see Figure 2). Obviously, both peptides accumulate as a result of a series of pathophysiological parameters detrimental to survival. Our results indicate that cut points for the diagnosis of acute heart failure or for the prediction of mortality established in cohorts of patients not recruited because of end-stage renal disease (ESRD) do not necessarily apply for ESRD patients. In the present study MR-proANP and MR-proADM values above these cut-off points were demonstrated in a very high proportion of patients which might not only reflect the hemodynamic disturbances and risk prediction. Both peptides are produced in the kidney with important biological functions for the kidney. A compensatory increase in concentrations as well as a reduced clearance of both peptides requires searching for suitable cut points in patients with ESRD. Interestingly, we observed in the present study not only higher cut points of the two peptides but also a combination of both parameters to be predictive for outcomes.

Presumably, ANP reflects the central volume overload and intrinsic heart disease, whereas ADM reflects the decompensated reaction to the multifactorial stress state in preserving the integrity of the cardiovascular system in ESRD (22). It is of interest that we observed a strong association with all-cause and cardiovascular mortality but not with cardiovascular events combining fatal and non-fatal events. The slight increase in risk for CVD events (HR = 1.45, 95%CI 0.87–2.44) if both peptides were in the upper tertile was driven by the fatal CVD events. If these fatal events were excluded, the estimates for non-fatal CVD events did not show in any direction (HR = 1.04, 95%CI 0.45–2.39). This underscores that the pronounced increases of both peptides might reflect more the hemodynamic disturbances related to cardiac dysfunction rather than atherosclerotic processes resulting in non-fatal CVD events without hemodynamic decompensation. The combined measurement of these two peptides might provide surrogate variables for volume status but also act as an early indicator of deteriorating hemodynamic changes.

The diagnostic and prognostic utility of MR-proADM has been shown in various non-CKD cohorts and for different endpoints such as heart failure, cardiovascular events, and all-cause mortality [15]–[18]. In hemodialysis patients plasma ADM levels were associated with clinical conditions such as cardiac dysfunction, excessive blood volume and systemic inflammation but also with cardiovascular outcomes and mortality [13], [24]. There is strong evidence that MR-proADM is produced in the kidney to exert hemodynamic actions on renal function [4]. That might be an explanation why MR-proADM was one of the strongest predictors for the progression of early stages of kidney impairment, which was even independent from baseline GFR measured by iohexol clearance [6], [7].

It is currently unclear whether the systemic and renal haemodynamic effects are caused by MR-proADM itself or predominantly through its active peptide ADM since an active role of MR-proADM is currently unclear. However, the eligible measurement of the active hormone ADM was doubted largely mainly due to the short half-life of 22 minutes and the partial binding to complement factor H [25]–[27]. Since MR-proADM and ADM are stoichiometrically generated and MR-proADM is more stable [28], MR-proADM seems to be at least a reliable surrogate marker for the active hormone ADM. The same holds true for MR-proANP and ANP. A perfect correlation between the midregional parts of the prohormones and the respective active hormones can not be expected since differences in the metabolic rates are assumed. Nevertheless, the midregional parts of the prohormones seem to mirror the hard to measure active hormones, otherwise the manifold associations with clinical endpoints would not be observed.

Our findings complement and extend previously reported cohort studies of end-stage renal disease for the biomarkers ANP [8]–[12] and ADM [13] (Table 5). Most of these studies were done in small cohorts and/or short observation periods and only one study considered ADM. Our cohort differs from these earlier studies since we investigated baseline levels in patients before first dialysis treatment. It therefore avoids a potential survival bias caused by the higher mortality rates during the first year of dialysis treatment which are not considered in cross-sectional cohorts or mixed cohorts of prevalent and incident dialysis patients.

**Table 5:** Prospective studies in dialysis patients investigating the association between atrial natriuretic peptide (ANP) and adrenomedullin (ADM) on clinical outcomes.

Study	Design	Follow-up	Endpoint and number of patients with endpoint	HR (95% CI)
**Atrial natriuretic peptide (ANP)**				
**Zoccali et al.**2001 [11]	Cohort study: 246 patients with end-stage renal disease without heart failure	26 mos.	All-cause mortality: 63CV mortality: 35	All-cause mortality: 2.39 (1.59–3.59) for ln ANP adjusted for Kt/V, age, ln albumin, ln cholesterol, diabetes.4.22 (1.79–9.92) for patients in the 3^rd^ vs. the 1^st^ tertile of ANP.CV mortality: 2.13 (1.29–3.52) for ln ANP adjusted for calcium, Kt/V, age.3.80 (1.44–10.03) for patients in the 3^rd^ vs. the 1^st^ tertile of ANP.
**Goto et al.**2002 [12]	Cohort study: 53 hemodialysis patients	11.3 mos.	Cardiac events: 13	118±21 vs. 56±5 pg/mL in patients with compared to without cardiac events.
**Nakatani et al.**2003 [8]	Cohort study: 105 hemodialysis patients	24 mos.	Cardiac death: 11	32 (4–252) for ANP>50 pg/mL vs. <50 pg/mL.3.5 (1.6–7.4) for ln ANP (adjusted for LVMI and CRP).
**Odar-Cederlöf et al.**2003 [9]	Cohort study: 33 hemodialysis patients	47 mos.	All-cause mortality: 18Early deaths (<1 year): 6	All-cause mortality: ANP (predialysis) 1.004 (1.000–1.007);ANP (postdialysis): 1.006 (1.000–1.012).Early deaths: ANP (predialysis) 1.007 (1.001–1.013);ANP (postdialysis) 1.006 (0.995–1.016).
**Yoshihara et al.**2005 [13]	Cohort study: 67 hemodialysis patients	1 yr.	7 patients died and 8 CV events	Mortality and CV events combined: 1.41 (0.36–5.56) for ANP≥230 (median) compared to ANP<230.
**Rutten et al.**2006 [10]	Cohort study: 68 peritoneal dialysis patients	1.5–4.5 yrs.	All-cause mortality: 10	11.3 (1.4–91.9) for ANP>median compared to ANP<median.7.9 (0.9–72.1) adjusted for age, comorbidity, residual GFR.
**This study**	Incident cohort study: 201 dialysis patients	56 mos.	All-cause mortality: 86CV mortality: 37	All-cause mortality: 1.44 (1.17–1.78) per SD increase of ANP.CV mortality: 1.75 (1.28–2.39) per SD increase of ANP.
**Adrenomedullin (ADM)**				
**Yoshihara et al.**2005 [13]	Cohort study: 67 hemodialysis patients	1 yr.	7 patients died and 8 CV events	Mortality and CV events combined: 4.55 (1.23–16.80) for ADM≥4.55 (median) compared to ADM<4.55.
**This study**	Incident cohort study: 201 dialysis patients	56 mos.	All-cause mortality: 86CV mortality: 37	All-cause mortality: 1.32 (1.11–1.58) per SD increase of ADM.CV mortality:1.41 (1.10–1.82) per SD increase of ADM.
**Combination of the upper tertiles of ANP and ADM compared to other tertiles**				
**This study**	Incident cohort study: 201 dialysis patients	56 mos.	All-cause mortality: 86CV mortality: 37	All-cause mortality: 2.82 (1.76–4.53) per SD increase of ANP.CV mortality: 3.30 (1.62–6.70) per SD increase of ANP.

CV, cardiovascular; SD, standard deviation.

MR-proANP concentrations were substantially increased in our cohort of dialysis patients when compared to patients with mild to moderate impairment of kidney function [6] as well as to a high risk cohort of heart failure patients [14]. While the role of ANP as regulator of the cardiovascular system is established, its physiological regulatory role on transport processes in the nephron is under debate [5]. ANP has been known to be primarily produced in the cardiac atrium, however, up-regulation of ANP mRNA expression has been demonstrated in extra-atrial tissues such as the kidneys [29]. The exact pathophysiological significance of kidney-synthesized ANP has not been defined yet.

To date prognostic biomarkers which predict outcome in this high risk cohort of CKD patients are sparse but highly demanded. However, as we discussed recently [6] the measurement of the active hormones has little clinical utility due to the short half life of most of the bioactive peptides, their immediate binding to receptors [27], their interaction with binding proteins [30] and several technical difficulties [30]. This is even more important when long-term stored samples are analysed because the active hormones undergo degradation even in frozen samples which is not the case for their propeptides. Therefore, replacing the problematic measurement of bioactive rapidly cleared peptides by measuring the non-functional, stable peptides MR-proANP and MR-proADM derived from their precursors represents a valuable advance for clinical practice. Interestingly, proteolytic degradation of pro-ANP seems to be mainly directed to the N- and C-terminal parts, whereas the midregion is significantly more stable [28] promoting this region to a suitable target of measurement. In addition, circulating mid-regional fragments are not influenced by a binding protein, making it suitable for immunometric analysis.

### Strengths and limitations of the study

The prospective recruitment of all patients starting dialysis treatment over a period of six years in a clearly described area allowed a complete recruitment of patients requiring renal replacement therapy with almost no loss to follow-up during a long observation period. We can therefore exclude the most important bias of cross-sectional studies with a mix of prevalent and incident cases and the resulting survival bias. We furthermore considered even the observation period following kidney transplantation by modeling the treatment status in a time-dependent model, but on the other hand excluded by sensitivity analysis, that this procedure has obscured our results.

There are also some limitations of this study. A first limitation is the relatively small number of patients and events. We therefore could only adjust our analyses for a small number of variables (6 variables in Model 1). An extended adjustment with more variables (Model 2 and additional sensitivity analyses) was provided but should only be considered as sensitivity analysis. Interestingly, the estimates for MR-proADM and MR-proANP remained widely stable in Models 1 and 2, the additional sensitivity analyses and a very simple model only adjusted for age and sex. This argues for a pronounced independence of the two investigated variables for the prediction of endpoints. Second, we examined patients at the time immediately before renal replacement therapy was started. Therefore, some patients might not have been in a steady metabolic or extracellular volume state of the parameters evaluated. Concentrations of these hormones might therefore not necessarily be applicable to other cohorts of patients with CKD. On the other hand, values measured after the start of dialysis treatment as done in other mostly smaller studies might represent dialysis dose or dialysis efficacy and thus answer a different question. Third, our study includes only Caucasian patients of a clearly described geographical region with almost complete ascertainment of incident dialysis patients over a defined period of time. It therefore lacks generalizability to other ethnic populations as well as other recruitment procedures. Finally, since the measurements of the two peptides are not rigorously standardized, concentrations measured by various assays are not necessarily comparable.

### Conclusion

Among incident dialysis patients MR-proANP and MR-proADM were shown to be associated with all-cause mortality and cardiovascular mortality, with the highest risk when both parameters were in the upper tertiles.

## Supporting Information

Table S1 The association of MR-proANP and MR-proADM as well as MR-proANP tertiles and MR-proADM tertiles and furthermore for patients with high risk (both MR-proANP and MR-proADM in the highest tertile) with different endpoints using multiple Cox-proportional hazards models.
(PDF)
