# Supplementary material for: The Association of Mid-Regional Pro-Adrenomedullin and Mid-Regional Pro-Atrial Natriuretic Peptide with Mortality in an Incident Dialysis Cohort
Source: PLoS One. 2011 Mar 7;6(3):e17803. doi: 10.1371/journal.pone.0017803 (PMC3049793; doi:10.1371/journal.pone.0017803)
Supplement: Table S1 — The association of MR-proANP and MR-proADM as well as MR-proANP tertiles and MR-proADM tertiles and furthermore for patients with high risk (both MR-proANP and MR-proADM in the highest tertile) with different endpoints using multiple Cox-proportional hazards models. (PDF) [file pone.0017803.s001.pdf]

Table S1: The association of MR-proANP and MR-proADM as well as MR-proANP tertiles and MR-proADM tertiles and furthermore for patients with high risk (both MR-proANP and MR-proADM in the highest tertile) with different endpoints using multiple Cox-proportional hazards models.

	**All-cause mortality** (n events = 81)			**Cardiovascular mortality*** (n events = 36)			**Cardiovascular disease**** (n events = 83)			**Peripheral arterial disease***** (n events = 53)		
	HR	95%CI	p-value	HR	95%CI	p-value	HR	95%CI	p-value	HR	95%CI	p-value
**MR-proANP (per 1 SD increase)[a]**												
Model 1	1.45	(1.17, 1.80)	0.001	1.74	(1.27, 2.38)	<0.001	1.15	(0.92, 1.45)	0.224	1.30	(0.98, 1.72)	0.065
Model 2	1.36	(1.06, 1.73)	0.014	1.72	(1.22, 2.43)	0.002	1.06	(0.82, 1.37)	0.641	1.31	(0.95, 1.79)	0.096
**MR-proADM (per 1 SD increase)[a]**												
Model 1	1.31	(1.10, 1.57)	0.003	1.41	(1.10, 1.81)	0.008	1.16	(0.97, 1.39)	0.095	1.07	(0.82, 1.41)	0.607
Model 2	1.24	(1.01, 1.52)	0.039	1.43	(1.07, 1.92)	0.015	1.11	(0.90, 1.36)	0.327	1.08	(0.80, 1.46)	0.627
**MR-proANP (tertiles)[b]**												
≤522 pmol/L	1			1			1			1		
523-794 pmol/L	0.97	(0.49, 1.91)	0.926	1.52	(0.49, 4.69)	0.464	0.95	(0.52, 1.76)	0.876	0.87	(0.39, 1.95)	0.741
≥795 pmol/L	1.72	(0.90, 3.32)	0.103	2.90	(0.96, 8.73)	0.059	1.09	(0.58, 2.04)	0.792	1.02	(0.46, 2.28)	0.966
**MR-proADM (tertiles)[b]**												
≤2.40 nmol/L	1			1			1			1		
2.41-3.10 nmol/L	1.14	(0.59, 2.19)	0.693	1.06	(0.39, 2.83)	0.911	1.02	(0.58, 1.80)	0.932	1.11	(0.56, 2.20)	0.763
≥3.11 nmol/L	2.60	(1.41, 4.80)	0.002	3.09	(1.24, 7.67)	0.015	1.55	(0.89, 2.72)	0.122	1.35	(0.66, 2.75)	0.412
**MR-proANP- MR-proADM- Score[b]**												
Low & intermediate risk	1			1			1			1		
High risk	2.95	(1.80, 4.83)	<0.001	3.57	(1.72, 7.39)	0.001	1.49	(0.88, 2.49)	0.134	1.58	(0.84, 2.95)	0.154

MR-proANP, mid-regional pro-atrial natriuretic peptide; MR-proADM, mid-regional pro-adrenomedullin.

Model 1: adjusted for age, sex, previous CVD**, diabetes mellitus, time-dependent type of renal replacement therapy.

Model 2: adjusted as in model 1 and additionally for albumin, CRP, smoking, native fistula, echocardiography (ejection fraction ≤60% and >60%).

[a] For MR-proANP and MR-proADM 1 standard deviation (SD) increment was 524 pmol/L and 1.29 nmol/L, respectively. One SD was taken as the unit of increment for each of the continuous outcome variables to ensure comparability of Hazard Ratios.

[b] Adjusted for age, sex, previous CVD**, diabetes mellitus, time-dependent type of renal replacement therapy.

* **CV mortality**: myocardial infarction, heart failure, sudden cardiac death, ischemic stroke, hemorrhagic stroke.

** **CVD**: myocardial infarction, percutaneous transluminal coronary angioplasty, aortocoronary bypass, angiographically-proven coronary stenosis ≥50%, ischemic or hemorrhagic cerebral infarction, transient ischemic attack, carotid stenosis and carotid endarterectomy.

*** **PAD**: significant ultrasound- or angiographically-proven vascular stenosis, percutaneous transluminal angioplasty, peripheral bypass, amputation.
